# The CHARMS pilot study: a multi-method assessment of the feasibility of a sexual counselling implementation intervention in cardiac rehabilitation in Ireland

**DOI:** 10.1186/s40814-018-0278-4

**Published:** 2018-07-02

**Authors:** Patrick J. Murphy, Chris Noone, Maureen D’Eath, Dympna Casey, Sally Doherty, Tiny Jaarsma, Andrew W. Murphy, Martin O’Donnell, Noeleen Fallon, Paddy Gillespie, Amirhossein Jalali, Jenny Mc Sharry, John Newell, Elaine Toomey, Elaine E. Steinke, Molly Byrne

**Affiliations:** 1 Health Behaviour Change Research Group, School of Psychology, NUI Galway, Galway, Ireland; 2 Disciplines of Occupational Therapy and Radiation Therapy, School of Medicine, Trinity College Dublin, Dublin, Ireland; 3 School of Nursing and Midwifery, NUI Galway, Galway, Ireland; 4 Department of Psychology, Royal College of Surgeons in Ireland, Dublin, Ireland; 5 Department of Social and Welfare Studies, Linköping University, Linköping, Sweden; 6 Department of General Practice, NUI Galway, Galway, Ireland; 7 HRB Clinical Research Facility, NUI Galway, Galway, Ireland; 8 Cardiac Rehabilitation Unit, Tallaght Hospital, Dublin, Ireland; 9 School of Business and Economics, NUI Galway, Galway, Ireland; 10 School of Mathematics, Statistics, and Applied Mathematics, NUI Galway, Galway, Ireland; 11 School of Nursing, Wichita State University, Kansas, USA; 12 Room 2.77, Discipline of Occupational Therapy, Trinity Centre for Health Sciences, St. James’s Hospital, Dublin 8, Ireland

**Keywords:** Behaviour change, Implementation intervention, Complex intervention, Cardiac rehabilitation, Cardiovascular disease, Sexual counselling, Feasibility study, Pilot trial

## Abstract

**Background:**

Many people living with cardiovascular disease (CVD) are affected by sexual problems associated with the condition. International guidelines recommend all patients with CVD should receive sexual counselling, yet this is rarely provided by health professionals. The current study piloted the Cardiac Health and Relationship Management and Sexuality (CHARMS) intervention, a complex multi-level intervention designed to increase the implementation of sexual counselling guidelines in hospital-based cardiac rehabilitation (CR) in Ireland.

**Methods:**

The CHARMS intervention, consisting of awareness training and skills development for staff, and education and support for patients, was implemented in two CR centres. Following a repeated measures design, quantitative and qualitative feasibility, fidelity, cost, and outcome data were collected from staff and patients at baseline (T1, pre-intervention), at 3 months post-baseline (T2, post-intervention), and at 6 months post-baseline (T3, post-intervention). Data were organised according to a 14-point reporting framework of methodological issues that should be examined in pilot and feasibility studies. To inform a future definitive trial, potential solutions to identified feasibility issues were generated using the ADePT process for decision-making after pilot and feasibility trials.

**Results:**

Most elements of the study protocol were executed smoothly, and intervention implementation was successful. Patients’ (*N* = 42) responses to the intervention were positive. The reporting framework and the ADePT process facilitated the identification of two overarching feasibility problems, as well as solutions to be implemented in a definitive trial: (1) a high level of patient attrition in the pilot study, to be addressed through the use of financial incentives, reducing the length of the patient questionnaire, and providing a telephone survey option; and (2) negative staff perceptions, to be addressed through an augmented staff intervention, reframing ‘sexual counselling’ as ‘sexual education and support’ to fit with professional role perceptions, and reviewing all intervention terminology with a CR staff member to ensure acceptability.

**Conclusions:**

This article reports the successful piloting of a novel sexual counselling implementation intervention in cardiac rehabilitation. The utilisation of an extended reporting framework and the ADePT process facilitated the identification of adaptations necessary to ensure the feasibility of a definitive trial, thereby maximising methodological transparency.

**Electronic supplementary material:**

The online version of this article (10.1186/s40814-018-0278-4) contains supplementary material, which is available to authorized users.

## Background

Cardiovascular disease (CVD) is already the most common cause of morbidity and mortality globally [1], and prevalence is projected to increase substantially [2]. Many people living with CVD are affected by sexual problems associated with the condition, which negatively affect quality of life, psychological wellbeing, and relationship satisfaction [3, 4]. The Cardiac Health and Relationship Management and Sexuality (CHARMS) baseline study, conducted by the current research group, investigated the presence and treatment of sexual dysfunction in cardiac rehabilitation (CR) in Ireland [5–8]. This study revealed high rates of sexual dysfunction among CR patients, a desire to spend more time discussing sexual issues with healthcare providers, infrequent receipt of treatment, and low satisfaction with service provision in this area [7].

According to guidelines endorsed by the American Heart Association and the European Society of Cardiology, sexual counselling should be provided to all patients with CVD as part of CR [9], with sexual counselling defined as ‘an interaction with patients that includes information on sexual concerns and safe return to sexual activity, as well as assessment, support, and specific advice related to psychological and sexual problems’. Despite these guidelines, and the expressed preferences of patients, health professionals surveyed in the CHARMS baseline study reported rarely discussing sexual issues with patients, and lacking awareness, knowledge, and confidence in this area [6, 7].

Informed by these results, the CHARMS intervention was developed using the Behaviour Change Wheel framework [10]. It is a complex, multi-level intervention aimed at the implementation of sexual counselling guidelines in CR, thereby improving sexuality-related outcomes for patients with CVD. The process of intervention development and details of intervention content have been reported previously [11]. Intervention components are summarised below in the “Methods” section.

### The current study

The current study examined the feasibility of a definitive cluster randomised controlled trial of the CHARMS intervention, with a focus on the following research questions:

1. What is the likely effect size for the primary outcome among patients?
2. What is the optimal number of CR centres (clusters) and CR patients (participants) needed to sufficiently power a definitive trial?
  - What are the recruitment and attrition rates for clusters and participants?
  - What is an unbiased estimate of the intraclass correlation coefficient (ICC) for the primary outcome among participants?
3. Is the intervention both feasible and acceptable?
  - Are the intervention components acceptable to staff and patients?
  - Are the outcome measures acceptable to staff and patients?
  - Will the intervention be delivered as designed (i.e., can fidelity be assured)?

To ensure a focus on methodological issues rather than outcomes, we adopted an extended reporting framework which includes 14 methodological issues that need to be examined in feasibility research [12]. To inform a future definitive trial, potential solutions to identified feasibility issues were generated using a process for decision-making after pilot and feasibility trials (ADePT) [13]. The ADePT process entails: (1) categorising feasibility issues according to whether they affect the trial only, real world implementation only, or both; (2) listing potential solutions according to whether they would change aspects of the intervention, the trial design, or the intervention context; (3) assessing potential solutions in terms of possible effectiveness and feasibility of implementation; and (4) selecting solutions based on evaluations of effectiveness, feasibility, cost effectiveness, and how solutions could be applied concurrently in a definitive trial.

## Methods

This section briefly describes the study protocol detailed elsewhere [14]. Adaptations to this protocol enacted during the execution of the pilot study are described in the “Results” section.

### Design

This study is a multicentre feasibility study [15], with the CHARMS intervention implemented in two CR centres in Ireland. The intervention was implemented at both the staff level and the patient level (see below). Following a repeated measures design, quantitative and qualitative acceptability, feasibility, fidelity, and outcome data were collected from staff and patients at baseline (T1, pre-intervention), at 3 months post-baseline (T2, post-intervention), and at 6 months post-baseline (T3, post-intervention).

### The intervention setting

The CHARMS intervention was integrated into phase III hospital-based CR programmes, which are usually 6 to 8 weeks in duration and delivered to patients within 6 weeks of discharge from acute care [16].

### Eligibility criteria

CR centres which delivered phase III CR to at least 80 patients per year were deemed eligible. Eligible patients were enrolled in phase III CR at a participating centre, were over the age of 18, and had CVD.

### Sample size calculation

A sample size calculation with the Sexual Self-Perception and Adjustment Questionnaire (SSPAQ) as the primary outcome indicated that 30 patients should be recruited in each centre [14].

### Recruitment procedures

#### Centre recruitment

All 37 CR centres listed in the directory of the Irish Association of Cardiac Rehabilitation were invited to express interest in participation [17]. Interested centres were assessed for eligibility and stratified according to location (Dublin, non-Dublin). Within each stratum, it was intended that centres would be approached in random order until one centre was successfully recruited.

#### Staff recruitment

Recruitment materials were distributed to staff by the CR coordinators. Participating staff posted completed materials directly to the researchers.

#### Patient recruitment

It was intended that the CR coordinators would post a recruitment pack (containing an invitation letter, information sheet, consent form, questionnaire, and opt-out form) to patients enrolling in CR. Patients who wished to participate could return consent forms and questionnaires to the research team by post. The patient recruitment pack also included a partner recruitment pack, containing the same materials with just slight modifications in wording. Patients could give this to their partner, who could then choose to participate in the study with the patient.

### The CHARMS intervention

The CHARMS intervention was implemented at both the staff level and the patient level. Details of the intervention, including behaviour change techniques (BCTs), are available elsewhere [11].

#### The CHARMS staff intervention

A CHARMS educator (co-author NF) delivered a 2-h training session to CR staff, focusing on intervention rationale, sexual counselling guidelines and skills development, delivery of the patient intervention, and creating an implementation plan for the patient intervention.

#### The CHARMS patient intervention

CR staff delivered a group-based education session to patients, focusing on CVD and sexuality, communication strategies, cardiac risks associated with sexual activity, tips for resuming sexual activity, and invitations to request one-to-one consultations if desired. The content of the session was detailed in an information booklet provided to all patients. An awareness-raising poster was displayed in participating centres.

### Data collection

Data were intended to be collected from staff, patients, and partners at T1, T2, and T3. Measures included in the staff, patient, and partner questionnaires are summarised in Table 1.

**Table 1:** Summary of outcome measures for cardiac rehabilitation staff, patients, and partners

	Number of items	T1	T2	T3^*a*^	Source
Staff-specific measures					
Demographic and professional information	7	x			
Sexuality-related practice	8	x	x	x	[1]
Knowledge, confidence, and awareness	3	x	x		[1]
Sexual attitudes and beliefs	12	x	x		[2]
Capability, opportunity, and motivation	10	x	x		
Patient- and partner-specific measures					
Demographic and medical information	12	x	x		
Sexual Self-Perception and Adjustment Questionnaire	28	x	x		[3]
Health-related QoL (EQ-5D-5L)	5	x	x		[4]
Cardiovascular-specific health-related QoL (HeartQoL)	14	x	x		[5]
Sexual activity and sexual problems	4	x	x		[1]
Depression (PHQ-2)	2	x	x		[6]
Relationship satisfaction (ENRICH)	15	x	x		[7]
Satisfaction with services	3		x		
Staff, patient, and partner measures					
Barriers to discussing sexual problems	17	x	x		[1]
Sex after myocardial infarction knowledge test	25	x	x		[8]
Comment/feedback (including time to completion)		x	x	x	

SSPAQ, primary outcome measure

^a^Due to time constraints, T3 data were not collected from patients and partners

### Evaluation of the pilot

#### Quantitative evaluation of feasibility and acceptability

Researchers documented recruitment and attrition rates, as well as missing data in questionnaires. All questionnaires included a feedback section, inviting comment on comprehensibility, acceptability, possible improvements, and completion time.

#### Qualitative evaluation of feasibility and acceptability

Staff and patients consenting to the qualitative component of the study were interviewed twice, initially focusing on early experiences of the intervention and the study as a whole, and later focusing on changes in opinions and experiences over time. Patients had the option of inviting their partners to be interviewed with them. Interviews were semi-structured. All were audio-recorded, transcribed, and entered into NVivo 10 [18]. A qualitative content analysis was conducted [19].

#### Fidelity

For the staff intervention, the CHARMS educator documented the elements of the intervention delivered, staff attendance, and duration. The staff intervention was also audio recorded and coded for the presence of intervention elements and BCTs. For the patient intervention, CR staff documented the elements of the intervention delivered, patient attendance, and duration. Patients completed exit questionnaires assessing the elements of the intervention delivered, and quality of delivery. Data from qualitative interviews were used to elucidate fidelity findings.

#### Health economics

A cost-outcome description was undertaken. With respect to costs, data were collected on resource use related to intervention delivery, and patient resource use in relation to sexual function medications and other services. With respect to outcomes, the economic analysis focused on quality adjusted life years (QALYs) estimated using the EQ-5D-5L questionnaire and value set [20].

## Results

The methodological issues addressed, a mapping of these issues to the research questions specified in the published protocol [14], relevant findings, and evidence are summarised in Table 2 and described in turn below. A completed CONSORT extension for pilot and feasibility trials checklist is provided in Additional file 1.

**Table 2:** Summary of findings for 14 methodological issues addressed in feasibility research

Methodological issue	RQ	Findings	Evidence
1. Did the study allow a sample size calculation for the definitive trail?	1, 2, 2b	Although dependent on a small sample, sample size calculations were conducted	Adjusting for an estimated attrition rate of 75%, 88 participants should be recruited in each of 22 clusters (*n* = 1936)
2. What factors influenced eligibility and what proportion of those approached were eligible?	2a	Some CR centres approached were ineligible due to treating fewer than the minimum number of patients. All staff and patients were considered eligible.	2 out of 22 centres approached were ineligible.
3. Was recruitment successful?	2a	Centre and staff recruited proceeded smoothly. Revised strategy for patient recruitment proved successful.	See data on recruitment and attrition rates in Table 6.
4. Did eligible participants consent?	2a	Consent was obtained successfully.	See data on recruitment and attrition rates in Table 6.
5. Were participant successfully randomised and did randomisation yield equality in groups?		Not applicable to the current study	
6. Were blinding procedures adequate?		Not applicable to the current study.	
7. Did participants adhere to the intervention?	3c	Adherence to the staff and patient interventions was good	Fidelity measures showed intervention elements and BCTs generally delivered as intended
8. Was the intervention acceptable to participants?	3a	Staff had reservations about the sexual nature of the intervention. Patient perceptions of the intervention were highly positive.	70.6% of patients were very or somewhat satisfied with the education session. Qualitative evidence was supportive.
9. Was it possible to calculate intervention costs and duration?		Staff costs and intervention resource costs were estimated. A full cost-outcome analysis could not be conducted.	Staff costs: €1296 per year Patient booklets: €200 per year Training: €400
10. Were outcome assessments completed?	3b	All staff outcome assessments were completed as intended. The patient outcome assessments were modified in response to staff concerns, and T3 patient data were not collected due to time constraints.	See summary of outcome data in Table 11.
11. Were outcome measured those that were the most appropriate outcomes?		Outcome measures were appropriate. However, the patient questionnaire was perceived to be lengthy and repetitive, and staff had concerns about its content	See qualitative data reported in section 11 of the Results
12. Was retention to the study good?	2a	Centre and staff retention was good. Patient retention was problematic.	See data on recruitment and attrition rates in Table 6.
13. Were the logistics of running a multicentre trial assessed?		Yes. Patient recruitment and assessment in a future definitive trial was identified as being resource intensive.	The patient recruitment process derived in this study depended on one-to-one introductions, and a large sample size has been estimated for a definite trial
14. Did all components of the protocol work together?		All intervention components, and their assessment, worked together as intended and integrated smoothly within the existing CR programmes.	Reported problems in the various processes were minimal.

### 1. Did the study allow a sample size calculation for the main trial?

A linear mixed model analysis was fitted to estimate the ICC at baseline in order to estimate the degree of within-cluster homogeneity induced by the design. The model considered the effect of gender, age, and education on the primary outcome (SSPAQ) scores at T1 and included a random effect for centre (the model is summarised in Tables 3 and 4). The ICC was estimated to be less than .0001, suggesting that individuals within clusters were no more similar than those from different clusters. However, it is plausible that some variability due to clustering is likely in a definitive trial that includes multiple clusters; therefore, an upper bound of .05 is a reasonably conservative estimate of the ICC to adopt in this instance.

**Table 3:** Fixed effects of the explanatory variables on SSPAQ scores at T1 (*N* = 42)

	Estimate	Std. Error	*t* value
Intercept	− 10.74	28.41	− 0.378
T1 SSPAQ	0.76	0.13	5.965*
Gender	10.38	5.83	1.779
Age	− 0.04	0.31	− 0.130
Education	3.55	1.31	2.721

*p < .05

**Table 4:** Random effect of centre on SSPAQ scores at T1 (*N* = 42)

	Variance	SD
Centre (intercept)	< 0.0001	< 0.01
Residual	350.2	18.71

Summary statistics and 95% confidence intervals were calculated for the change score for the primary outcome (SSPAQ), for all patients and disaggregated by centre, gender, and education level (Table 5). Examination of the confidence intervals indicates that the change scores for all patients and for all disaggregations were equivalent to zero, albeit based on a small sample.

**Table 5:** Mean differences, standard deviations, and 95% confidence intervals in SSPAQ change scores, T1 to T2 (*n* = 22)

	Mean difference	Standard deviation	95% confidence interval
All participants	− 1.10	13.07	(− 7.15, 4.95)
Centre			
Dublin	− 5.50	15.55	(− 18.50, 7.50)
Non-Dublin	1.75	10.87	(− 5.16, 8.66)
Gender			
Male	1.13	13.36	(− 6.26, 8.53)
Female	− 8.00	10.42	(− 20.93, 4.93)
Education			
Primary	− 12.50	16.78	(− 39.21, 14.21)
Secondary	− 4.50	3.54	(−36.21, 27.27)
Post-secondary	8.67	20.82	(− 43.04, 60.38)
Third level or higher	0.89	8.98	(− 6.01, 7.79)

This study was focused on feasibility issues, and as such was not designed to detect a significant effect of the intervention. Nonetheless, from examining the current data, it would be appropriate to design any definitive trial to detect a difference in mean improvement of 5 units, arguably the smallest effect size of clinical importance. Based on an estimated standard deviation for the change in SSPAQ scores of 13 units observed here, and assuming an ICC of .05, this would require a minimum of 11 clusters randomly allocated to each arm, each containing 22 patients (*n* = 484 in total), in order to have 80% power at the 5% significance level. Adjusting for an estimated 6-month attrition rate of 75% (see below), 88 patients should be recruited in each of the 22 clusters (*n* = 1936).

### 2. What factors influenced eligibility and what proportion of those approached were eligible?

Of the 37 rehabilitation centres contacted, 22 (61.1%) expressed interest in the study, 5 of which were in Dublin (from a total of 8), and 17 of which were outside Dublin (from a total of 27). One centre outside Dublin indicated they were not interested in the study due to staffing constraints. The remaining 14 centres (38.8%) did not respond to study invitations. All 5 interested centres in Dublin were eligible, as were 13 of the 17 outside Dublin. All ineligible centres were treating fewer than the minimum number of patients required. All staff and patients were considered eligible.

### 3. Was recruitment successful?

The interested Dublin centres were approached in random order about participation in Spring 2016. The first two centres declined participation due to concerns about the sensitive sexual nature of the study and for practical reasons related to staff leave. The third centre approached agreed to participate. For centres outside Dublin, one centre was purposively recruited for the pragmatic reasons of size and location. Recruitment of staff in both participating centres proceeded smoothly.

The planned recruitment of patients by posting recruitment packs to them was deemed to be inappropriate by CR staff due to data protection concerns. A second strategy was devised, in which a researcher (PM) gave a 5-min study introduction to each CR group and distributed recruitment packs. This group-based strategy proved to be ineffective, so a third strategy was developed. Early in each CR programme, patients were invited by a member of staff to a private meeting with a researcher (PM). At this meeting (lasting approximately 10 min), the study was explained and patients were offered a recruitment pack. Those who took the pack were phoned by the researcher at an agreed time within 2 to 3 working days. In this follow-up call, patients could ask questions and indicate if they would like to participate. Participating patients were asked to return a signed consent form and completed questionnaire, and those who declined were encouraged to return an opt-out form. This third strategy resulted in a 40.4% (*N* = 42) recruitment rate among eligible patients. The target of 30 patient participants per centre would have been reached were it not for study time constraints. Recruitment and attrition rates are presented in Table 6. Patient recruitment commenced in June 2016 and ceased in December 2016, to allow sufficient time for follow-up assessments before the scheduled termination of the study in March 2017.

**Table 6:** Recruitment and attrition rates among staff and patients

	Eligible	T1 (consent)	T2	T3	Recruitment rate	Attrition rate
Staff	13	6	6	6	46.1%	0%
Patients	104	42	22	n/a	40.4%	47.6%

*n/a* not applicable

### 4. Did eligible participants consent?

Several staff in both centres indicated that staff non-consent was due to a lack of time, rather than any concerns about the intervention itself. However, in both centres one member of staff who had not formally consented still took part in the staff intervention and aided in the delivery of the patient component. Demographic and professional data for participating staff members at T1 are shown in Table 7.

**Table 7:** Demographic and professional data for participating staff in both centres (*N* = 6)

	Dublin centre (*n* = 3)		Non-Dublin centre (*n* = 3)	
	*M* (*SD*)	*n*	*M* (*SD*)	*n*
Age	51.3 (4.7)		46.7 (9.6)	
Gender				
Male		1		1
Female		2		2
Duration of employment	6.5 (8.2)		12.0 (8.0)	
Profession				
Cardiac coordinator		1		1
Clinical nurse specialist		1		
Occupational therapist				1
Physiotherapist				1
Social worker		1		

**Fig. 1:**
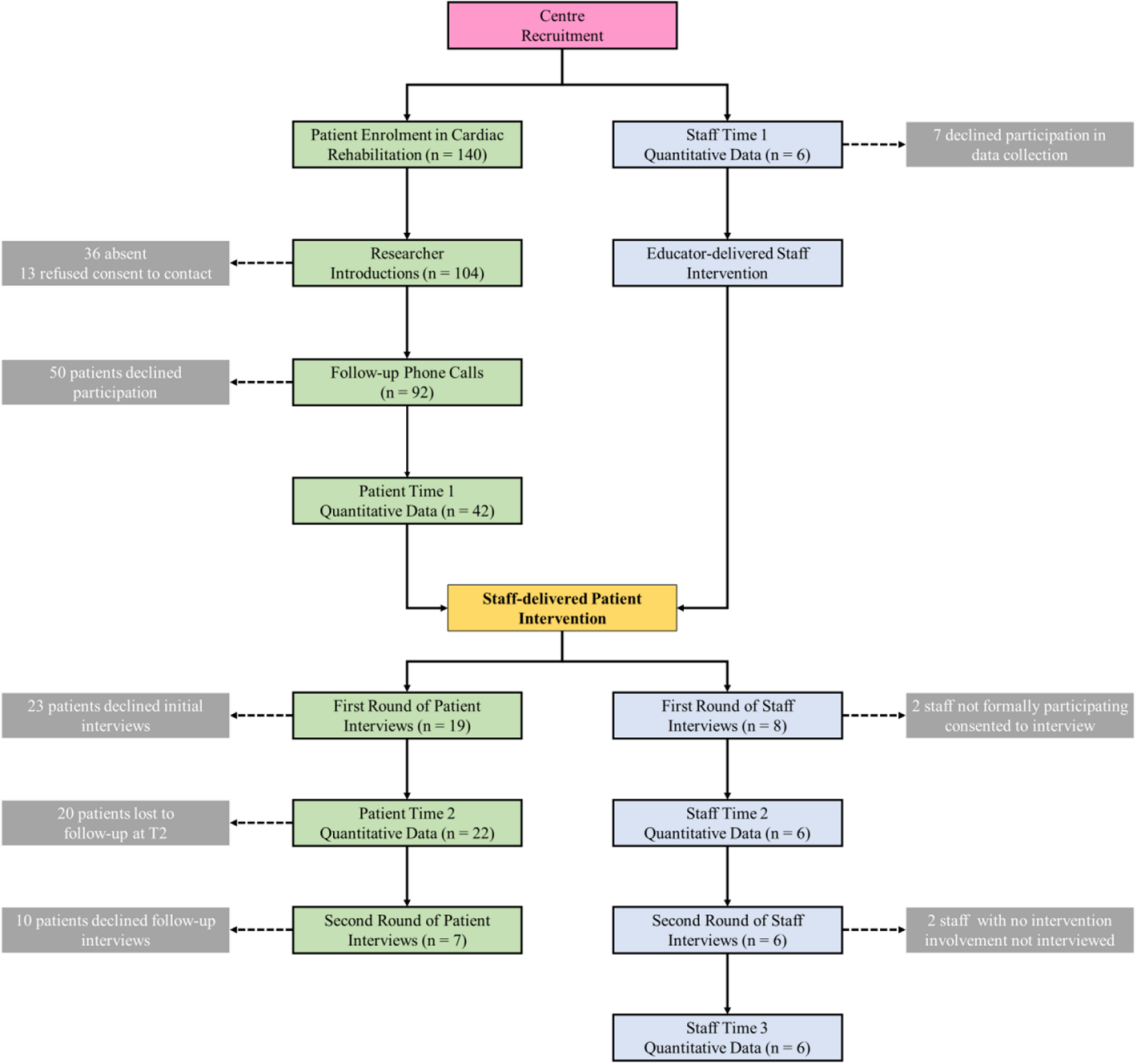
Flow diagram of the progress through the CHARMS pilot study for both staff (in blue) and patients (in green)

Just over 40% (*n* = 42) of eligible patients consented to participate (see Table 6). For participating patients at T1, demographic characteristics are shown in Table 8, medical characteristics in Table 9, frequency of reports of sexual problems in Table 10, and descriptive statistics for outcome measures in Table 11. A flowchart depicting staff and patient engagement with the study is shown in Fig. 1.

**Table 8:** Demographic characteristics for participating patients at T1 (*N* = 42)

	Dublin centre (*n* = 19)		Non-Dublin centre (*n* = 23)	
	Men	Women	Men	Women
Gender	18 (94.7%)	1 (5.3%)	18 (78.3%)	5 (21.7%)
Age (*M* (*SD*))	58.83 (9.8)	60 (n/a)	62.3 (13.2)	61.8 (7.8)
Use of tobacco products				
Yes	0 (0%)	0 (0%)	2 (11.1%)	0 (0%)
No	18 (100%)	1 (100%)	16 (88.9%)	5 (100%)
Education				
No formal education	0 (0%)	0 (0%)	2 (11.1%)	0 (0%)
Primary only	3 (16.7%)	1 (100%)	2 (11.1%)	0 (0%)
At least some secondary	6 (33.3%)	0 (0%)	8 (45.5%)	1 (20%)
At least some tertiary	9 (50%)	0 (0%)	5 (27.8%)	4 (80%)
Sexual Orientation				
Heterosexual	18 (100%)	1 (100%)	18 (100%)	5 (100%)
Homosexual	0 (0%)	0 (0%)	0 (0%)	0 (0%)
Bisexual	0 (0%)	0 (0%)	0 (0%)	0 (0%)
Other	0 (0%)	0 (0%)	0 (0%)	0 (0%)
Relationship Status				
Married/civilly partnered/cohabiting	12 (66.7%)	1 (100%)	15 (83.3%)	2 (40%)
Widowed/separated/divorced	2 (11.1%)	0 (0%)	0 (0%)	1 (20%)
In a relationship, but live alone	1 (5.6%)	0 (0%)	0 (0%)	0 (0%)
Single	2 (11.1%)	0 (0%)	1 (5.6%)	1 (20%)
Other	1 (5.6%)	0 (0%)	2 (11.1%)	1 (20%)

*Note*: *p* values for group differences are subject to a Bonferroni correction to account for multiple comparisons (*α* criterion = .002). No significant differences were found

**Table 9:** Medical characteristics for participating patients at T1 (*N* = 42)

	Dublin centre (*n* = 19)		Non-Dublin centre (*n* = 23)	
	Men (*n* = 18)	Women (*n* = 1)	Men (*n* = 18)	Women (*n* = 5)
Cardiac procedures				
Stenting (angioplasty or PTCA)	13 (72.2%)	1 (100%)	16 (88.9%)	3 (60%)
Bypass graft (coronary artery bypass graft)	3 (16.7%)	0 (0%)	3 (16.7%)	0 (0%)
Cardiac and other medical conditions				
Acute coronary syndrome	0 (0%)	0 (0%)	1 (5.6%)	0 (0%)
Angina	2 (11.1%)	1 (100%)	3 (16.7%)	1 (20%)
Anxiety	1 (5.6%)	0 (0%)	0 (0%)	1 (20%)
COPD	0 (0%)	0 (0%)	2 (11.1%)	1 (20%)
Coronary heart disease	5 (27.8%)	1 (100%)	8 (44.4%)	2 (40%)
Depression	2 (11.1%)	0 (0%)	1 (5.6%)	1 (20%)
Diabetes	2 (11.1%)	0 (0%)	0 (0%)	0 (0%)
Heart attack	4 (22.2%)	0 (0%)	9 (50%)	4 (80%)
High blood pressure	8 (44.4%)	1 (100%)	7 (38.9%)	3 (60%)
High cholesterol	12 (66.7%)	0 (0%)	8 (44.4%)	1 (20%)
Prostate cancer	0 (0%)	n/a	0 (0%)	n/a
Stroke	1 (5.6%)	0 (0%)	0 (0%)	0 (0%)
Use of sexual performance medications	1 (5.6%)	0 (0%)	3 (16.7%)	0 (0%)
Cardiovascular medications				
Anti-coagulants/blood thinners	7 (38.9%)	1 (100%)	9 (50%)	2 (40%)
Antiplatelet agents	15 (83.3%)	0 (0%)	17 (94.4%)	5 (100%)
ACE inhibitors	3 (16.7%)	0 (0%)	5 (27.8%)	3 (60%)
Angiotension II receptor blockers	3 (16.7%)	1 (100%)	2 (11.1%)	0 (0%)
Beta blockers	14 (77.8%)	1 (100%)	13 (72.2%)	3 (60%)
Calcium channel blockers	1 (5.6%)	0 (0%)	1 (5.6%)	0 (0%)
Cholesterol-lowering medications/statins	15 (83.3%)	1 (100%)	17 (94.4%)	3 (60%)
Digitalis preparations	0 (0%)	0 (0%)	0 (0%)	0 (0%)
Diuretics	1 (5.6%)	0 (0%)	2 (11.1%)	2 (40%)
Other	4 (22.2%)	1 (100%)	9 (50%)	0 (0%)

*Note*: *p* values for group (centre) differences are subject to a Bonferroni correction for multiple comparisons (*α* criterion = .002). No significant differences were found

*n/a* not applicable

**Table 10:** Reports of sexual problems at T1 (*N* = 42)

	Dublin centre (*n* = 19)		Non-Dublin centre (*n* = 23)	
	Men (*n* = 18)	Women (*n* = 1)	Men (*n* = 18)	Women (*n* = 5)
Lacked interest in sex	6 (33.3%)	1 (100%)	7 (38.8%)	3 (60%)
Did not find sex pleasurable	6 (33.3%)	1 (100%)	3 (16.6%)	3 (60%)
Were unable to come to orgasm	9 (50%)	1 (100%)	2 (11.1%)	2 (40%)
Felt anxious about performance	5 (27.7%)	1 (100%)	5 (27.7%)	2 (40%)
Came to orgasm too quickly (*men only*)	5 (27.7%)	n/a	4 (22.2%)	n/a
Trouble maintaining an erection (*men only*)	7 (38.8%)	n/a	7 (38.8%)	n/a
Pain during intercourse (*women only*)	n/a	1 (100%)	n/a	4 (80%)
Trouble becoming lubricated (*women only*)	n/a	1 (100%)	n/a	4 (80%)

*n/a* not applicable

**Table 11:** Descriptive statistics (*M*, *SD*) for the study measures for patients at T1 (*N* = 42) and T2 (*n* = 22)

	Dublin centre (*n* = 19)				Non-Dublin centre (*n* = 23)			
	Men (*n* = 18)		Women (*n* = 1)		Men (*n* = 18)		Women (*n* = 5)	
	T1	T2	T1	T2	T1	T2	T1	T2
SSPAQ total	103.13 (17.15)	96.17 (15.18)	117.00 (−)	− (−)	98.07 (30.29)	96.14 (41.07)	102.25 (24.05)	104.00 (20.07)
Anxiety	28.94 (4.75)	31.75 (3.84)	35.00 (−)	25.00 (−)	28.75 (7.51)	27.75 (10.04)	29.00 (5.16)	29.66 (5.03)
Depression	26.70 (5.07)	26.55 (2.83)	31.00 (−)	27.00 (−)	26.00 (6.98)	25.87 (10.74)	27.75 (4.86)	26.33 (4.16)
Self-efficacy	23.05 (6.81)	19.37 (9.07)	21.00 (−)	− (−)	22.80 (8.73)	24.00 (10.66)	23.25 (6.66)	25.33 (6.65)
Satisfaction	22.11 (7.42)	15.29 (7.54)	30.00 (−)	21.00 (−)	23.00 (9.55)	22.43 (12.34)	22.25 (6.99)	22.66 (8.50)
EQ-5D-5L	0.88 (0.15)	0.87 (0.13)	0.94 (−)	0.86 (−)	0.87 (0.14)	0.91 (0.11)	0.79 (0.12)	0.82 (0.14)
QALYS	–	.212 (.042)	–	.226 (−)	–	.214 (.038)	–	.204 (.033)
HeartQoL	23.94 (9.55)	21.83 (7.60)	18.00 (−)	− (−)	22.67 (6.49)	22.80 (9.01)	30.00 (5.29)	26.00 (7.00)
PHQ-2	0.75 (1.00)	0.37 (0.74)	0.00 (−)	0.00 (−)	1.17 (1.59)	0.89 (1.36)	1.40 (0.89)	1.25 (0.96)
ENRICH	51.12 (9.22)	47.76 (14.28)	47.10 (−)	− (−)	44.20 (8.99)	53.34 (12.16)	44.94 (24.06)	58.56 (−)
SMIKT	62.11 (5.09)	65.00 (5.41)	63.00 (−)	62.00 (−)	62.36 (4.05)	61.43 (3.69)	63.25 (5.85)	65.00 (6.00)

### 5. Were participants successfully randomised and did randomisation yield equality in groups?

Randomisation of participants was not relevant in the current study.

### 6. Were blinding procedures adequate?

Blinding procedures were not relevant in the current study.

### 7. Did participants adhere to the intervention

#### Adherence to the staff intervention

At the non-Dublin centre, 4 staff attended the staff intervention and 1 did not. At the Dublin centre, 4 staff attended and 2 did not. Checklists and coded audio recordings indicated that all elements of the staff intervention were delivered in both centres. All planned BCTs were delivered in both centres, with the exception of a single BCT (“problem solving”) which was not effectively delivered in the Dublin centre.

#### Adherence to the patient intervention

In the non-Dublin centre, the patient education session was labelled on the programme timetable as ‘Return to Activities’, and patients were not pre-informed about the content. In the Dublin centre, the session was labelled as ‘Resuming Sexual Activity’, patients were explicitly pre-advised about the content and told that attendance was optional. Despite this variation, there was no statistically significant difference in attendance rates between the non-Dublin centre (69.5%, *n* = 85) with the Dublin centre (69.1%, *n* = 71), indicating that knowing the sexual content of the session was not a deterrent to attendance.

Fidelity data indicated that all elements of the patient education session were delivered as intended in the Dublin centre, but two elements of the patient education session (emotional challenges, and coping as a couple) were consistently *not* delivered in the non-Dublin centre. These two elements had been assigned to a member of staff who withdrew support shortly after the intervention had been implemented. To minimise burden on the remaining staff members, the research team decided not to reassign these elements of the intervention, but to instead document the deviations from the protocol and carefully monitor fidelity for the remaining elements of the intervention. Across centres, approximately two thirds of patients rated each element of the education session as ‘very effectively’ delivered. The booklets were distributed at 100% of sessions, and posters were displayed in several locations in both centres.

### 8. Was the intervention acceptable to participants?

#### Acceptability of the staff intervention

Staff (*n* = 8) views of intervention acceptability were assessed via interviews after the patient intervention had been delivered a number of times (see Fig. 1). Some CR staff found it valuable to come together as a team to focus on the topic of sexuality during the CHARMS staff intervention, as busy schedules rarely allowed such opportunities. Several spoke of an increased awareness of sexual dysfunction following the training:

The training was very beneficial and I’m far more aware of [sexual dysfunction] now. I’m far more aware of what I should do…before it was ‘go back to your GP’ and it was like ‘shoo’…Whereas now I'm more inclined to follow it up, say okay you have come to me with this let’s talk about it and try to make sure it’s followed up on. Like take the reins….So no, it has made a huge difference, it’s subtle but I think it’s significant. (Centre 1, Staff 3)

While the value of the intervention was questioned by some CR staff who had extensive prior experience in dealing with sexual issues, it was particularly welcomed by those who did not have such experience, to a degree that the intervention would remain part of their practice even after the study ended:

…the information is good, it goes across to the patients well and I’m definitely going to continue with it…no matter what happens with the research I wouldn’t go back, it has to continue...I’ll never not include it. (Centre 2, Staff 5)

Recommendations for improvement were given, including a greater focus on the content of the patient education session and the content of the patient questionnaires.

When CR staff (*n* = 6) practice was assessed at T1 (pre-intervention), 3 reported that sexual issues were not addressed at all, and 3 reported that they were addressed but not effectively. When assessed at T2 (post-intervention, *n* = 6), 4 staff reported that sexual issues were addressed somewhat or very effectively.

#### Acceptability of the patient education session

Some CR staff had generalised concerns about the sexual nature of the CHARMS intervention and how it would be received by patients, fearing it could prompt a withdrawal from the rehabilitation programme:

And I think there has been some concern that maybe it might put people off coming into the sessions. (Centre 1, Staff 2)

However, in interviews carried out with patients (*n* = 19) post-intervention (see Fig. 1), most characterised the education sessions very positively, feeling the group setting was appropriate for the content as it did not put pressure on individuals to talk about themselves:

…there was no pressure on anything and information came out…I felt that the group session was a very effective method of getting the information across (Centre 2, Patient 10)

Some patients reported that the session normalised sexual problems and would make asking for help in the future easier:

So once you start talking about something you find uncomfortable to talk about, the next time…it’s a little less difficult to open up. So I think for those people it would have been the most beneficial because it…opened the door to that discussion (Centre 1, Patient 10)

Several patients also spoke about the unexpected but very welcome benefit of realising they were not alone in living with sexual problems:

Believe it or not, it does take a weight off your shoulders because you know you are not the only one. (Centre 1, Patient 5)

Of the 17 patients who participated at T2, 12 were very or somewhat satisfied with the education session, 4 were neither satisfied nor dissatisfied, and only 1 was somewhat dissatisfied. No patient indicated that they were very dissatisfied. There was no significant difference in satisfaction across centres.

#### Acceptability of the patient booklet

The patient booklet was very favourably reviewed by CR staff who considered it to be well laid out, accessible, and informative:

I think the booklet is fantastic, great booklet, really like it. I think it’s great, I think it’s excellent and the posters are excellent…very informative…learned myself. I think they’re very good…they’re concise, they’re bright, they’re lovely, perfect. (Centre 2, Staff 1)

Staff members reported that patients seemed to be interested in the CHARMS booklet:

I left them up on the desk when the talk was finished and I was interested to see a lot of people went up, I would say the majority went up and took a book. (Centre 1, Staff 3)

Many patients commented positively on the booklet and were not inclined to offer any suggestions for improvement. However, one patient and his partner argued that the red colour and the explicit title would limit the number of people who would choose to pick it up, and suggested that the older age of the couple featured on the front cover excluded younger people like themselves.

#### Acceptability of the awareness raising poster

Comments about the poster were generally positive from both staff and patients, finding it a useful addition within CR:

It’s good because, I suppose, it gives people a chance to ask you … it’s there and it’s clear and it’s obvious and we have one in the gym as well (Centre 1, Staff 1)

### 9. Was it possible to calculate intervention costs and duration?

Healthcare resource usage by patients was minimal. At T2 no patient reported the use of medications to improve sexual function. Only 1 patient reported accessing GP and outpatient services but did not indicate frequency of attendance. Statistically significant improvements in EQ-5D-5 L scores were recorded among patients between T1 and T2. These responses were combined to estimate QALYs gained over the follow up period (see Table 11).

It is estimated that delivery of the CHARMS intervention would on average require 1 h of staff input per CR group, resulting in staff costs of €26.25 per CR group, based on the midpoint of the Health Service Executive salary scales for clinical nurse specialists [21]. Additional intervention costs would include patient booklets (€1.00 per booklet) and training costs (once off, estimated at €400).

### 10. Were outcome assessments completed?

#### Staff outcome assessments

Quantitative data were collected from staff at T1, T2, and T3 as intended. For staff, missing data at the item level was minimal. Questionnaire completion was relatively quick, requiring on average 13 min at T1 and 22 min at T2 (the T3 staff questionnaire was highly abbreviated, so time to completion was not assessed).

#### Patient outcome assessments

CR staff voiced strong concerns about the patient questionnaire, particularly about the perceived invasiveness of the sexuality-related measures:

they’re very intimate questions …. I have never seen such intimate questions asked of patients… I do think that they are inappropriately intrusive (Centre 1, Staff 1)

Staff reservations were sufficiently strong that in order to retain both centres in the study, the research team agreed to modify the patient questionnaires prior to distribution by removing the International Index of Erectile Function [22] and the Female Sexual Function Index [23].

The amended patient questionnaire required on average 31 min to complete at T1 and 31 min to complete at T2. Unfortunately, due to study time constraints, T3 data were not collected from patients. The proportion of missing data for patients at the item level for most scales (including the SSPAQ) was acceptable at less than 10% [24]. The measure with the highest proportion of missing data was the Barriers to Discussing Sexual Problems scale (10.9% at T2). A commonly cited reason for non-responses to this scale was discomfort with judging the performance of CR staff.

### 11. Were outcomes measured those that were the most appropriate outcomes?

The outcomes measured appear to have been appropriate for the intended purpose, but will require adjustment in a definitive trial.

A key term utilised in this study was ‘sexual counselling’. While CR staff did not object to performing the activities included in the definition of term ‘sexual counselling’ (see above), they did object to adopting the term itself as it appeared to conflict with their professional role identities:

I think ‘sexual counselling’ is too strong a term to use…We are not sexual counsellors in cardiac rehab and are not qualified in ‘sexual counselling’ (Centre 2, Staff 1)

Some CR staff also felt the patient questionnaire was biased towards producing criticism of them and created an unfair burden of care:

[P]utting all the onus on cardiac rehab to provide sexual counselling for the patients and if cardiac rehab didn’t do it then patients get a bad service … I don’t think that it should be put solely in the lap of cardiac rehab. (Centre 2, Staff 2)

It was suggested that greater involvement from CR staff in the development of the questionnaires would have been beneficial:

Maybe if somebody from cardiac rehab…had been involved in formulating those questions, I think they would have been different, somebody actually working on the floor (Centre 2, Staff 1):

Patients, however, pointed out that the expressed focus of the CHARMS study on sexuality would prepare patients for the nature of the questions:

…you know there’s going to be an intrusive element but not necessarily an invasive element. There’s no point filling out a survey unless you are prepared for the idea difficult questions need to be asked…(Centre 2, Patient 5)

Two patients identified that they benefitted from completing the questionnaire as it had enhanced their insight into their own difficulties:

You can’t find the problem if you don’t ask those type of questions…I wouldn’t have known necessarily that I had a problem if I didn’t answer the questions that way. (Centre 1, Patient 18)

However, the questionnaire was considered to be very repetitive, and some felt there was an ulterior motive behind that repetition:

It was sneaky. Yeah, it kept asking the same question. I noticed the same question 4 or 5 times, just worded differently. (Centre 2, Patient 10)

### 12. Was retention to the study good?

No attrition was observed among centres or staff. As shown above in Table 6, however, the attrition rate among patients observed from T1 to T2 was 47.6%. Approximating the attrition rate between time points at 50%, the projected attrition from T1 to T3 in a definitive trial would be 75%. This projected attrition rate has been incorporated into the sample size calculations reported above.

### 13. Were the logistics of running a multicentre trial assessed?

A full definitive trial would require significant researcher-input to deliver the staff intervention to centres in the intervention arm, and to manage the recruitment, assessment, and retention of both staff and patients in both arms of the trial at all time points. This is particularly the case given the contact-intensive method of patient recruitment developed in this study and the large estimated patient sample size required (*n* = 1936).

### 14. Did all components of the protocol work together?

All protocol components, modified where necessary as described, worked together and integrated smoothly within the existing CR programmes.

## Discussion

Most elements of the study protocol were executed smoothly, and intervention implementation was successful. The positive patient reactions were particularly welcome, given the prior concerns about the intervention’s sensitive nature. However, the reporting framework facilitated the identification of a number of feasibility problems relating to (1) patient attrition and (2) negative staff perceptions.

### Patient attrition

The attrition rate among patients observed here, and the projected attrition rate of 75% in a definitive trial, would render such a trial *unfeasible* within CR in Ireland. Attrition may have been exacerbated by the length and repetitiveness of the patient questionnaire. The ADePT process produced three solutions to be applied concurrently: (1) offering financial incentives for questionnaire returns, a strategy shown to be effective in a Cochrane review of recruitment and retention strategies [25]; (2) reducing the length and repetitiveness of the questionnaire by removing overlapping and non-essential measures; and (3) providing a telephone survey option, which proved effective in the CHARMS baseline research [7]. See Additional file 2 for further details.

### Negative staff perceptions

Negative perceptions held by staff included concerns that the sexual nature of the intervention might contribute to patient dropout from the CR programme, concerns that the patient questionnaire was too invasive because of its sexual content, and the perceived unsuitability of the term ‘sexual counselling’. Although caution among staff regarding any new intervention and possible deleterious consequences is wholly appropriate, it should be emphasised that those concerns were allayed in the current study. The ADePT process produced the following solutions: (1) augmenting the staff intervention with data from the current study, showing that patients had positive perceptions of the intervention; (2) replacing the term ‘sexual counselling’ with the term ‘sexual education and support’, which may be more acceptable to CR staff, and fits well with the actual process of providing care for sexual problems; and (3) review all aspects of the intervention with a CR professional to ensure terminology is acceptable. See Additional file 3 for further details.

### Limitations

The current data were gathered from relatively small self-selecting samples of staff and patients, who may have been particularly open to engaging with sexual issues. Therefore caution in generalisation is warranted.

The logistics of running a larger multicentre trial have been acknowledged, and this will require close attention. Procedures for randomisation and for managing centres in the control arm will need to be carefully developed.

There were challenges in the applying the reporting framework [12] and the ADePT process [13]. For example, distinguishing between recruitment (point 3 of the framework) and consent (point 4) was problematic, as patients were deemed to be recruited if they consented. As another example, it was unclear what level of evidence should be acceptable in the ADePT process. The original authors appear to accept expert opinion, but whether this is sufficient is questionable. There is limited guidance available in the literature, and methodological advances in this area are needed.

## Conclusion

This article has reported the successful piloting of a novel sexual counselling implementation intervention in cardiac rehabilitation. The reporting framework and the ADePT process facilitated the identification of adaptations necessary to ensure the feasibility a definitive trial. This article is therefore a valuable addition to the research literature, providing an exemplar of methodological transparency in piloting and feasibility work.

## Additional files

Additional file 1: CHARMS CONSORT Extension Checklist. (DOC 226 kb)

Additional file 2: Application of ADePT process to patient attrition. (DOCX 19 kb)

Additional file 3: Application of ADePT process to staff perceptions. (DOCX 14 kb)

## Abbreviations

ADePT: A process for decision-making after pilot and feasibility trials
BCT: Behaviour change technique
CHARMS: Cardiac Health and Relationship Management and Sexuality
CR: Cardiac rehabilitation
CVD: Cardiovascular disease
ICC: Intraclass correlation coefficient
QALY: Quality-adjusted life year
SSPAQ: Sexual Self-Perception and Adjustment Questionnaire
