# Supplementary material for: The CHARMS pilot study: a multi-method assessment of the feasibility of a sexual counselling implementation intervention in cardiac rehabilitation in Ireland
Source: Pilot Feasibility Stud. 2018 Jul 2;4:88. doi: 10.1186/s40814-018-0278-4 (PMC6027553; doi:10.1186/s40814-018-0278-4)
Supplement: Supplementary file 2 — Application of ADePT process to patient attrition. (DOCX 19 kb) [file 40814_2018_278_MOESM2_ESM.docx]

| STEP 1: PROBLEM TYPE |
| --- |
| **TYPE A**: There was a problem with patient attrition observed in the pilot study. This problem is likely to impact a definitive trial only, not real world implementation. |
| **EVIDENCE**:   1. Of the patients who participated at T1 (*n* = 42), close to half (*n* = 20, 47.6%) were lost to follow-up at T2. Extrapolating to T3 would give an approximate attrition rate of 75% for a definitive trial. 2. The questionnaires may have required too long to complete for many patients (average 30 minutes at T1 and T2). 3. Repetition due to the inclusion of overlapping measures may have impacted acceptability, making questions appear ‘sneaky’. |

| STEP 2: SOLUTIONS |
| --- |
| **CHANGE ASPECTS OF:**  **a) INTERVENTION**  Not applicable  **b) TRIAL DESIGN**   1. Decrease participant burden by removing overlapping and non-essential measures from the patient questionnaires. Measures to be removed include the SMIKT (25 items), the HeartQOL (14 items), ENRICH (15 items), barriers to discussing sexual problems (17 items). It is estimated this would reduce time to completion from 30 minutes to 15-20 minutes, and also address the perceived repetitiveness of the patient questionnaire. 2. Offer small financial incentives (e.g., €10 vouchers) to patients for completing assessment at all time points. 3. Provide additional means of assessment completion, particularly telephone-based. (Web-based methods are not thought to be generally applicable with the target population of older adults).   **c) CONTEXT**  Not applicable |

| STEP 3: ASSESSMENT OF SOLUTIONS (TRIAL DESIGN) | | |
| --- | --- | --- |
| Could solution b1 be **effective** in a trial setting? **YES** | Could solution b2 be **effective** in a trial setting? **YES** | Could solution b3 be **effective** in a trial setting? **YES** |
| **EVIDENCE**: A Cochrane review of strategies to improve retention in randomised trials suggested that shorter questionnaires may be effective, but the evidence was inconclusive [1]. However, the CHARMS pilot study intentionally included overlapping, potentially redundant, measures, with the intention of assessing their acceptability in the target population. Removing overlapping and non-essential measures should address issues with the questionnaires raised by patient participants in the pilot study. | **EVIDENCE**: There is evidence from a Cochrane review that the provision of even small financial incentives effectively increases the return of postal questionnaires [1]. | **EVIDENCE**: Although a Cochrane review reported no clear evidence that a telephone survey offered advantages over a postal survey [1], the CHARMS baseline research utilised a telephone survey which proved highly effective with the target population. |
|  |  |  |
| Could solution b1 be **feasible** in a trial setting? **YES** | Could solution b2 be **feasible** in a trial setting? **YES** | Could solution b3 be **feasible** in a trial setting? **YES** |
| **EVIDENCE**: Removing overlapping measures from the patient questionnaire could easily be achieved prior to a definitive trial. | **EVIDENCE**: The provision of monetary incentives is becoming increasingly common practice in trials [2, 3]. Given that financial incentives can help prevent the failure of a trial, their inclusion in trial budgets should be acceptable to potential funders. | **EVIDENCE**: The CHARMS research team has experience in conducting large scale telephone surveys, which can be costed and funded appropriately. |

| Step 4: Evaluation of Solutions |
| --- |
| BOX 1: OPTIONS THAT SHOULD WORK IN TRIAL CONTEXT |
| **Stage 1: Options (ranked by likely feasibility & effectiveness**   1. Financial incentives 2. Shorter questionnaires 3. Telephone surveys |
| **Stage 2: Potential to combine solutions**  Shorter questionnaires may be more practical to administer by telephone, but otherwise the solutions can be enacted independently. |
| **Stage 3: Most cost effective solutions**  The listed solutions are easily incorporated into the trial design, and it should be possible to fund them appropriately to ensure any definitive trial is successful. |

References

1. Brueton, V.C., et al., *Strategies to improve retention in randomised trials.* The Cochrane Library, 2013.

2. Bugge, C., et al., *A process for Decision-making after Pilot and feasibility Trials (ADePT): development following a feasibility study of a complex intervention for pelvic organ prolapse.* Trials, 2013. **14**(1): p. 353.

3. Hilton, P., et al., *A mixed methods study to assess the feasibility of a randomised controlled trial of invasive urodynamic testing versus clinical assessment and non-invasive tests prior to surgery for stress urinary incontinence in women: the INVESTIGATE-I study.* Trials, 2015. **16**(1): p. 400.
