# Supplementary material for: The CHARMS pilot study: a multi-method assessment of the feasibility of a sexual counselling implementation intervention in cardiac rehabilitation in Ireland
Source: Pilot Feasibility Stud. 2018 Jul 2;4:88. doi: 10.1186/s40814-018-0278-4 (PMC6027553; doi:10.1186/s40814-018-0278-4)
Supplement: Supplementary file 3 — Application of ADePT process to staff perceptions. (DOCX 14 kb) [file 40814_2018_278_MOESM3_ESM.docx]

| STEP 1: PROBLEM TYPE |
| --- |
| **TYPE B**: Negative staff perceptions of the sexual nature of the intervention and its assessment. This problem is likely to impact both a definitive trial, and real-world implementation. |
| **EVIDENCE**:   1. Staff members had concerns that the sexual nature of the intervention was unsuitable for some patients, and might contribute to patients dropping out of the CR programme 2. Staff members had concerns that the patient questionnaires were too invasive because of their sexual content, and would be likely to cause offence. 3. CR staff rejected the term ‘sexual counselling’ as it conflicted with professional role identities |

| STEP 2: SOLUTIONS |
| --- |
| **CHANGE ASPECTS OF**:  **a) INTERVENTION**  1) Augment the staff intervention with data from the current study showing that patients had positive perceptions of the intervention.  2) Replace the term ‘sexual counselling’ with the term ‘sexual education and support’, which may be more acceptable to staff involved in intervention delivery, and fits well with the actual process of providing care to patients for sexual problems.  3) Review all aspects of the intervention design and content with a CR professional to ensure adaptations are appropriate and acceptable.  **b) TRIAL DESIGN**  **c) CONTEXT** |

| STEP 3: ASSESSMENT OF SOLUTIONS (INTERVENTION DESIGN) | | |
| --- | --- | --- |
| Could solution a1 be **effective** in trial and real-world settings? **YES** | Could solution a2 be **effective** in trial and real-world settings **YES** | Could solution a3 be **effective** in trial and real-world settings **YES** |
| **EVIDENCE**: Augmenting the staff intervention as described should directly address staff concerns | **EVIDENCE**: Replacing the term ‘sexual counselling’ with the term ‘sexual education and support’ should directly address staff concerns. | **EVIDENCE**: A review with a CR professional should ensure that negative staff perceptions are addressed prior to a definitive trial. |
|  |  |  |
| Could solution a1 be **feasible** in trial and real-world settings? **YES** | Could solution a2 be **feasible** in trial and real-world settings? **YES** | Could solution a3 be **feasible** in trial and real-world settings? **YES** |
| **EVIDENCE**: The staff intervention can be readily adapted by the research team | **EVIDENCE**: Intervention materials can be readily adapted by the research team | **EVIDENCE**: The research team can carry out such a review with a CR professional as part of preparations for a definitive trial. |

| Step 4: Evaluation of Solutions |
| --- |
| BOX 1: OPTIONS THAT SHOULD WORK IN TRIAL AND REAL-WORLD SETTINGS |
| **Stage 1: Options (ranked by likely feasibility & effectiveness**   1. Augment staff intervention 2. Replace term ‘sexual counselling’ with ‘sexual education and support’ 3. Conduct review with CR professional |
| **Stage 2: Potential to combine solutions**  A review with a CR professional should complement solutions 1 and 2 above. |
| **Stage 3: Most cost effective solutions**  The listed solutions are easily incorporated into the intervention design, and it should be possible to fund them appropriately to ensure any definitive trial is successful. |
